# Analysis of a normalised expressed sequence tag (EST) library from a key pollinator, the bumblebee *Bombus terrestris*

**DOI:** 10.1186/1471-2164-11-110

**Published:** 2010-02-15

**Authors:** Ben M Sadd, Michael Kube, Sven Klages, Richard Reinhardt, Paul Schmid-Hempel

**Affiliations:** 1 Institute of Integrative Biology (IBZ), ETH Zürich, Universitätsstrasse 16, 8092 Zürich, Switzerland; 2 Max Planck Institute for Molecular Genetics, Ihnestrasse 73, 14195 Berlin, Germany

## Abstract

**Background:**

The bumblebee, *Bombus terrestris *(Order Hymenoptera), is of widespread importance. This species is extensively used for commercial pollination in Europe, and along with other *Bombus *spp. is a key member of natural pollinator assemblages. Furthermore, the species is studied in a wide variety of biological fields. The objective of this project was to create a *B. terrestris *EST resource that will prove to be valuable in obtaining a deeper understanding of this significant social insect.

**Results:**

A normalised cDNA library was constructed from the thorax and abdomen of *B. terrestris *workers in order to enhance the discovery of rare genes. A total of 29'428 ESTs were sequenced. Subsequent clustering resulted in 13'333 unique sequences. Of these, 58.8 percent had significant similarities to known proteins, with 54.5 percent having a "best-hit" to existing Hymenoptera sequences. Comparisons with the honeybee and other insects allowed the identification of potential candidates for gene loss, pseudogene evolution, and possible incomplete annotation in the honeybee genome. Further, given the focus of much basic research and the perceived threat of disease to natural and commercial populations, the immune system of bumblebees is a particularly relevant component. Although the library is derived from unchallenged bees, we still uncover transcription of a number of immune genes spanning the principally described insect immune pathways. Additionally, the EST library provides a resource for the discovery of genetic markers that can be used in population level studies. Indeed, initial screens identified 589 simple sequence repeats and 854 potential single nucleotide polymorphisms.

**Conclusion:**

The resource that these *B. terrestris *ESTs represent is valuable for ongoing work. The ESTs provide direct evidence of transcriptionally active regions, but they will also facilitate further functional genomics, gene discovery and future genome annotation. These are important aspects in obtaining a greater understanding of this key pollinator species.

## Background

Social insects, especially those belonging to the order Hymenoptera, have been an extremely successful group. They take on a wide diversity of roles around the world, and while some are considered as pests, others are seen as vital for ecosystem functioning and even commercial prosperity. Bumblebees (the genus *Bombus*) fall into the latter beneficial groups, being important members of natural pollinator assemblages and providing commercial pollination services [1].

Bumblebees have been an object of diverse scientific study due to their importance, behaviour, social life, and a number of other fascinating traits. To name but a few, bumblebees have been utilised in research on social evolution [2,3] and organisation [4,5], development [6,7], plant-pollinator interactions [8-10], learning [11,12], invasion biology [13], host-parasite ecology [14-17], ecological immunology [18-20], and community ecology [21,22]. While not officially domesticated, bumblebees still present an important agricultural resource with a significant economic load [23,24]. Bumblebees in both Europe (mainly *B. terrestris*) and North America (mainly *B. impatiens*) are bred commercially by the hundred thousands for this reason, with the colonies being used in the pollination of a variety of standard glasshouse crops [23]. Many have considered their economic importance to be on the rise given the recent declines in honeybee numbers [25]. Bumblebees not only provide valuable pollination services in the agricultural sector, but the pollination they carry out is an integral part of many natural ecosystems [26-28]. The importance of bumblebees comes sharply into focus when we consider the threat that pollinators currently face worldwide. This pollinator crisis has been epitomised by the recent honeybee colony collapse disorder [25], but the bumblebee has also suffered population declines in many areas over a number of decades [29-31].

Given their importance, a good knowledge of bumblebees from the genomic level up is of great interest. An improved set of genomic resources for bumblebees would facilitate further studies in the bumblebee, and comparisons with other bees such as the honeybee, *Apis mellifera*, where the genome is already sequenced [32]. There is currently a limited set of resources available for genetic investigation, and at the time of writing, for *B. terrestris*, there were 61 cDNA sequences and 288 nucleotide sequences (172 of which represent microsatellite markers) in NCBI Genbank (http://www.ncbi.nlm.nih.gov, November 2009). Further tools available include a linkage map for *B. terrestris *[33] and a BAC library [34].

In order to greatly expand the genomic resources available for *B. terrestris *we constructed a normalised expressed sequence tag (EST) library from thorax and abdomen tissue of workers. ESTs are short, unedited, randomly selected, single pass reads from cDNA libraries [35]. They provide evidence of transcriptionally active regions in an organism and are an excellent resource for upstream work including gene discovery, functional genomics and marker discovery [35,36]. Standard cDNA libraries can have problems with redundancy, and rare transcripts are often under-represented. Therefore, we employed normalisation in order to increase the number of unique transcripts discovered [37].

Here we describe a collection of 29,428 ESTs, which represent a valuable resource for further ecological and evolutionary studies in *Bombus *spp. and for comparative studies with other Hymenoptera and insects. Sequences have been deposited in the GenBank, EMBL, and DDBJ nucleotide sequence databases under the Accession numbers FN611035 through to FN640462. For further material, including consensus sequences of assembled contigs, please contact the authors.

## Results and Discussion

### Sequence assembly and features

A total of 29,428 quality controlled EST sequences were assembled using TGI clustering tools (TGICL). The assembly generated 4,682 contigs and 8,651 singletons. The average number of sequences per contig was 4.43 with the maximum being 35. Contigs averaged 960 bases in length with the longest contig being 3,542 bases. In total 13,333 unique sequences were produced. A table showing all EST and contig statistics can be found as an additional file (Additional file 1).

### Sequence annotation

The 13,333 unique sequences were used in a BLASTX search against the non-redundant (nr) database in Genbank (version dated 19th October 2009). A total of 7,844 (58.8%) ESTs had significant "hits" (cutoff = 1e-05) (Table 1 and Additional File 2). Of these, the majority (7,260 sequences) had "best-hits" to known sequences within the Hymenoptera, with a large proportion (6,296 sequences) of these belonging to *Apis *spp A total of 5,489 sequences (41.2%) had no significant similarity to any sequences contained in the nr database (Additional file 3). It is possible that many of these sequences without "hits" are cDNAs of known proteins, but consist mainly of untranslated regions (UTRs), therefore reducing the likelihood that matches are found through BLASTX. However, we cannot discount the possibility that some of the proteins are novel.

**Table 1:** Taxonomic distribution of the "best-hits" for the 13,333 post-assembly *Bombus terrestris *EST sequences (BLASTX, cutoff = 1e-05).

Taxonomic classification	Number of assembled ESTs
*Apis *spp.	6,296
*Nasonia vitripennis*	886
Existing *Bombus terrestris *sequences	8
Other *Bombus* spp.	49
Other Hymenoptera	21
Diptera	179
Coleoptera	110
Other Insecta	146
Other	149
No hits	5,489

Relevant groupings have been performed, but original counts on an individual taxon identifier level can be found as an additional file (Additional file 2).

### Gene ontology (GO) terms

The assembled *B. terrestris *ESTs were characterised for gene ontology terms in molecular function, biological process and cellular component by comparison against annotated proteins in the SwissProt and Trembl databases (UniProt Knowledgebase Release 15.9) using annot8r [38]. In order to increase the accuracy of any annotations, only GO terms that had been allocated by manual curation, and not those electronically inferred, were used. The distribution of the terms in each of the GO Slim overview categories can be seen in Table 2, and the full GO assignments can be found as an additional file (Additional file 4). Further, the same process was completed for predicted proteins in the *A. mellifera *and *Tribolium castaneum *genomes [32,39]. This allowed for a comparison to be made with the distribution of terms that we obtained for the *B. terrestris *unique sequences in this study. General patterns of distribution were similar between organisms, with limited exceptions where particular GO slim categories were over- or under- represented in the *B. terrestris *unique sequences (Table 2).

**Table 2:** Comparison of the percentage distribution of *Bombus terrestris *Gene Ontology terms (overview GO slim terms) based on the reported EST library and the distribution of Gene Ontology terms for all proteins predicted in each of the *Apis mellifera *and *Tribolium castaneum *genomes.

GO slim term*	***B. terrestris***	*A. mellifera*	***T. castaneum***
Molecular function			
motor activity	0.4% (14)	0.9%	0.8%
ligase activity	2.0% (64)	2.1%	2.0%
translation regulator activity	<0.1% (2)	<0.1%	<0.1%
signal transducer activity	1.6% (52)	2.7%	3.0% †
catalytic activity	11.8% (381)	10.4%	11.0%
binding	57.0% (1842)	57.8%	55.8%
transferase activity	9.8% (318)	8.6%	8.6%
transporter activity	4.0% (131)	5.6%	6.1% †
transcription regulator activity	2.5% (81)	3.3%	3.1%
antioxidant activity	0.1% (3)	0.1%	0.1%
lyase activity	0.7% (23)	0.7%	0.8%
oxidoreductase activity	5.2% (169)	3.7%	4.8%
isomerase activity	0.5% (17)	0.3%	0.3%
enzyme regulator activity	2.3% (73)	2.3%	2.0%
structural molecule activity	2.1% (67)	1.3%	1.6%
Biological process			
response to stimulus	7.0% (274)	5.9%	6.8%
cellular amino acid and derivative metabolic process	1.7% (66)	1.5%	1.7%
behavior	2.8% (111)	2.6%	2.7%
metabolic process	18.4% (718)	14.9% †	16.2%
cell differentiation	5.6% (220)	7.3%	7.1%
cell communication	6.1% (240)	8.1% †	8.3% †
nucleobase, nucleoside, nucleotide and nucleic acid metabolic process	10.2% (400)	6.9% †	6.5% †
extracellular structure organization	0.3% (10)	0.3%	0.2%
multicellular organismal development	10.1% (394)	12.0%	11.8%
cellular process	11.9% (467)	12.0%	11.3%
membrane fusion	0.2% (8)	0.2%	0.2%
transport	8.7% (342)	9.5%	9.7%
regulation of biological process	14.6% (573)	16.0%	14.9%
cell death	1.3% (52)	1.2%	1.4%
cell motion	0.9% (37)	1.4%	1.2%
Cellular component			
membrane	18.3% (472)	22.6% †	23.3% †
cell	6.6% (171)	7.6%	8.0%
intracellular	70.8% (1830)	66.0% †	63.6% †
extracellular	4.3% (111)	3.7%	5.1%

Actual counts appear in brackets after the percentage. Major categories of molecular function, biological process and cellular component are treated independently.

* Only manually curated terms were included to improve accuracy

† Also in bold. Statistically significant difference in the representation of the GO slim term between *Bombus *and the organism referred to in the column (p < 0.0014, Bonferroni-corrected Fisher's exact tests)

### Comparisons to the honeybee and other insects

The GC content of the *B. terrestris *unique sequences was calculated to be 36 percent (omitting ambiguous bases). Gene regions in the *A. mellifera *genome have a GC content of 29 percent, while in *D. melanogaster *and *A. gambiae *it is 44 and 47 percent, respectively [32]. The GC percentage based on these *B. terrestris *ESTs suggests that lower GC content of gene regions is not restricted to *Apis*, and may be a wider phenomenon. However, calculations of GC-content in *Nasonia vitripennis *mRNA (version dated 9th July 2007) at 46 percent hint that it is not Hymenopteran wide and further species would need to be studied to determine the exact range of this low GC-content.

**Figure 1:**
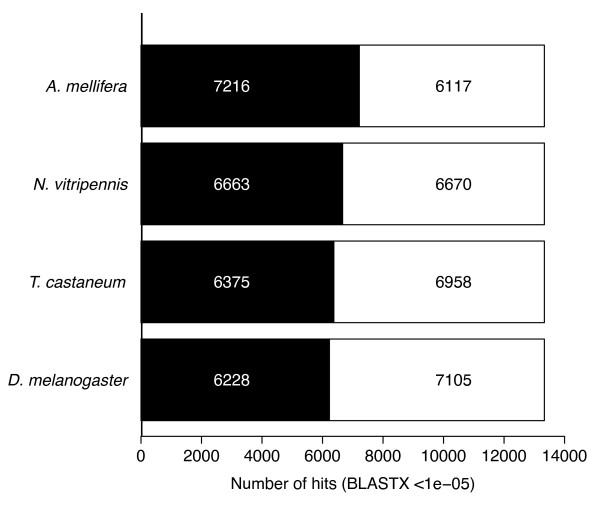
**The number of unique *Bombus terrestris *sequences uncovered in this project with significant "hits" (BLASTX, cutoff = 1e-05) to proteins predicted in each of the *Apis mellifera*, *Nasonia vitripennis*, *Tribolium castaneum *and *Drosophila melanogaster *genomes (black) and those with no "hits" to the predicted proteins of these organisms (white)**. Numbers inside bars represent the actual number of unique sequences.

We compared the *B. terrestris *unique sequences to proteins from genomes of each of four other insects, *A. mellifera*, *N. vitripennis*, *T. castaneum *and *D. melanogaster *(Figure 1, BLASTX, cutoff = 1e-05) (Additional file 5). The hierarchy of "hits" to each of these four insects reflects the evolutionary relationship between them and the bumblebee [32,40,41], with the closest related of the four, *A. mellifera*, giving 15.9 percent more "hits" than the most distantly realted, *D. melanogaster*. Despite this, 212 unique sequences from this EST project have significant "hits" to all of these insects except for *A. mellifera*. Potential explanations for this discrepancy include an incomplete annotation of genes in the *A. mellifera *genome or loss of genes or their protein coding capacity following the split from *Bombus *in the lineage leading to *Apis *(see below).

The 7,216 "hits" of unique sequences to *A. mellifera *proteins were to 4,480 unique *Apis *proteins. This level of redundancy suggests that 62 percent of the assembled sequences are actually derived from unique genes, yielding an estimation of 8,278 unique genes represented by this EST library. In addition to a comparison with the predicted proteins in the *A. mellifera *genome, the unique sequences derived from the *B. terrestris *EST library were also compared to the entire *A. mellifera *genome sequence (BLASTN, cutoff = 1e-08) [32]. A total of 10,024 unique sequences had "hits" to the genome, 6,749 of which already had "hits" to predicted *A. mellifera *proteins, but 3,275 which did not. These unique sequences with "hits" to the genome were distributed on the *Apis *linkage groups as follows, with the figure in brackets being the percent distribution of actual *Apis *protein coding sequences: 9.98 (9.57) percent on LG1, 5.89 (5.12) on LG2, 4.94 (4.61) on LG3, 4.94 (4.52) on LG4, 6.74 (5.77) on LG5, 3.73 (3.55) on LG6, 3.5 (3.56) on LG7, 5.25 (4.79) on LG8, 3.79 (3.76) on LG9, 5.04 (4.44) on LG10, 6.24 (5.97) on LG11, 3.20 (2.80) on LG12, 3.25 (3.03) on LG13, 4.03 (4.13) on LG14, 4.35 (4.61) on LG 15, 2.77 (2.30) on LG16, 0.02 (0.14) on the Mitochondria and 22.38 (27.34) on unassembled regions.

Particularly interesting is the apparent discrepancy between the number of *B. terrestris *unique sequences with matches to *A. mellifera *proteins and the number with matches to the *A. mellifera *genome. In fact, of the 212 unique sequences that had "hits" to all the other insect proteins tested but not *Apis*, 196 have "hits" to the *Apis *genome. There are a variety of possible reasons for a unique sequence having a "hit" to the *Apis *genome but not to predicted proteins from the same organism. The most obvious, is that the sequences in question are related to known *Apis *proteins, but they mainly contain untranslated regions (UTRs) and therefore do not produce significant "hits" in BLASTX alignments. When comparing the locations of the start of "hits" to the genome and the positions of predicted *Apis *protein coding sequences, 389 are either inside a protein coding sequence or span the boundary of one, suggesting that they comprise mostly of UTRs. Furthermore, 1,064 of the "hits" were within the range of possible *Apis *UTRs (maximum *Apis *UTR = 4,375 bases, obtained from the UCSC genome browser [42]). However, this still left 1,253 sequences, including 76 of the unique sequences that had "hits" to proteins from all the other insects tested but not *Apis*. These 1,253 sequences were further analysed for the presence of ORFs using ORF Finder http://www.ncbi.nlm.nih.gov/gorf. The median ORF size was 225 nucleotides (range 102-1,464) suggesting that many of the unique sequences have the potential to encode proteins. While some of these "hits" to the *Apis *genome may be spurious, it is possible that they are the result of either incomplete gene annotation in the *A. mellifera *genome or evolution of pseudogenes in the *Apis *lineage. In an attempt to gain a further insight into these possibilities, from the 20 unique sequences with the longest predicted reading frames we inspected more closely the sequences that had a highly significant "hit" to another insect protein (BLASTX, <1e-20) (Table 3). By "blasting" these proteins against the *Apis *genome (TBLASTN), we attempted to find reasons that these proteins are not in the set of predicted *Apis *proteins. All six of the proteins had "best-hits" in the same location of the genome as the unique sequences had, and visual inspection of alignments revealed premature stop codons were coded for in the *Apis *sequences in four out of the six cases.

**Table 3:** Unique sequences from the 20 longest ORFs that have "hits" to other insect proteins and also "hits" away from protein coding regions in the *Apis *genome, but are not part of the *Apis *protein set.

Project Identification (bom001no-)	Organism "best-hit"	Protein	Description	Potential reason for absence from *A. mellifera protein *set
CL2097Contig1	N. vitripennis	XP_001604128.1	similar to F-box protein 28	Stop codon
CL2223Contig1	N. vitripennis	NP_001123269.1	nucleoporin Ndc1	Unknown
CL2537Contig1	N. vitripennis	XP_001606900.1	similar to p53-like protein	Stop codon
CL2610Contig1	N. vitripennis	XP_001600663.1	similar to ENSANGP00000017887	Unknown
CL2809Contig1	N. vitripennis	XP_001604974.1	similar to GA18228-PA	Stop codon
P0115M15_F	P. humanus corporis	XP_002432715.1	nuclear pore complex protein nup98	Stop codon

Unique sequences, from the sequences with the 20 longest ORFs that have "hits" to the *Apis *genome at least 4'375 bases away from predicted *Apis *protein coding regions, with highly significant "best-hits" (BLASTX, 1e-20) to an insect protein. These "best-hit" insect proteins were "blasted" against the *Apis *genome (TBLASTN) and the resulting alignments scanned for potential elements hindering protein production.

### Immune genes and pathways

While the bumblebee workers used to produce this EST library were not immune challenged, the vast amount of work on immunity and host-parasite interactions in these insects [19,43-45] makes this category of genes nonetheless worthy of further interest. Furthermore, parasites have been implicated in declines of natural and commercial pollinators, such as bumblebees, worldwide [25,46,47], and thus knowledge of genes and pathways involved in immunity and parasite defence is highly important.

Based on the annotations of the unique sequences, and in comparison with proposed *A. mellifera *immune genes [48], we find that 134 unique sequences have "best hits" to proteins from genes characterised as immune related (Additional file 6). These 107 unique sequences in *Bombus terrestris *match to 67 *Apis *genes (a similar level of redundancy to that shown with all *A. mellifera *protein "hits"). In addition, based on the classification by Sackton *et al. *[49], 101 unique sequences have "best-hits" in *D. melanogaster *to proteins of 58 immune genes. However, a core set of only 32 unique sequences had both immune classified "hits" in *D. melanogaster *and *A. mellifera*, with "hits" to 24 and 25 unique genes, respectively. This could be due differential annotation of immune genes in the two organisms, or different divergence from *B. terrestris *among different genes.

**Figure 2:**
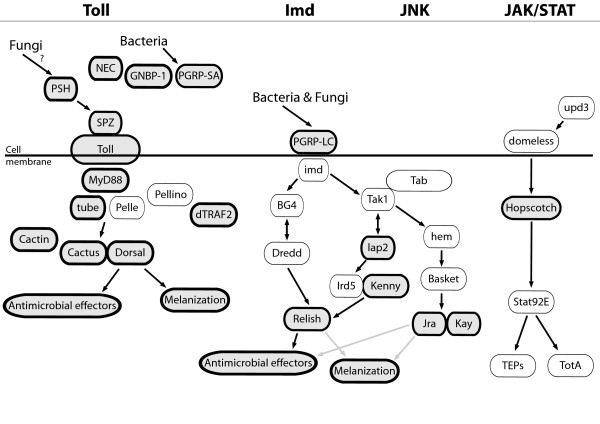
**Schematic showing the potential presence in bumblebees of components from the major described pathways in honeybee immunity that are implicated in the defence against bacterial, fungal and viral pathogens (schematic adapted from **[48]**)**. Shaded and bold outlined components indicate this component from either *Apis mellifera *or *Drosophila melanogaster *is "hit" by a sequence in the *Bombus terrestris *EST library.

The potential immune gene transcripts found span the major described pathways in the invertebrate immune system (Figure 2), and based on these certain components, we can begin to build a picture of immune defence in bumblebees. We find that there is evidence for the existence and transcription of integral genes in each of the described pathways.

### Potential EST-derived genetic markers

Traditional methods of isolating and characterising genetic markers can be expensive and time-consuming. However, ESTs can be a valuable resource for the initial identification of genetic markers that can subsequently be used in studies of molecular ecology [36]. Simple sequence repeats (SSRs) and single nucleotide polymorphisms (SNPs) allow population level studies both within the target species and related species.

Using MISA [50], a number of SSRs were identified in the unique sequences. We restricted our search for SSRs to di-, tri-, tetra-, penta-, and hexa- nucleotide motifs that were repeated at least six times for di-nucleotide and five times for all other motifs. We found a total of 589 markers found in 507 unique sequences. 397 di- (mean repeat length = 7.65, range = 6-17), 154 tri- (mean repeat length = 5.79, range = 5-17), 26 tetra- (mean repeat length = 6, range = 5-11), 6 penta- (mean repeat length = 5.83, range = 5-7), and 6 hexa- (mean repeat length = 6.33, range = 5-9) nucleotide repeats were found. 61 of the SSRs were found in compound formation with another (maximum of 100 nucleotides separation). While polymorphism remains to be verified for these markers with appropriate laboratory screens, previous levels of polymorphism in EST derived SSRs suggest that a large number of these markers will be variable [51].

These identified potential SSRs together with existing markers from *B. terrestris *[52-54] make up a significant resource for molecular ecology studies. While EST derived SSRs tend to show a lower level of polymorphism than those identified from genomic libraries, they can more often be used across related species [36,51,55]. Given the pervasive importance of species of the genus *Bombus *in temperate pollinator communities, this is a highly advantageous feature. In addition to SSRs, contigs built from a number of EST reads can be useful sources of potential SNPs. While the normalization procedure used in this work reduces such redundancy and thus the likelihood of finding SNPs, 1,973 contigs contain at least four sequences, a minimum for reliable identification of SNPs [56]. These contigs cover 2,124,958 bases. Using SNPidentifier [57] with minimum minor allele frequency of 0.1 and a minimum occurrence of 2, we were able to find 854 potential SNPs occuring in 573 contigs. These potential SNPs provide an additional set of genetic markers for subsequent population studies.

## Conclusions

The resource provided by the presented EST library will serve researchers in a diverse range of biological fields. The detected markers will facilitate further population level studies, and the sequences themselves can be used in investigations of particular genes, and also in functional genomic approaches. Further, as demonstrated here, the sequences allow genomic comparisons with the honeybee *A. mellifera *and other insects, informing on such things as potential gene loss or pseudogene evolution. This EST library will also be a practical resource in the assembly and annotation of the impending *B. terrestris *genome http://www.hgsc.bcm.tmc.edu. These elements, coupled with the major commercial and ecosystem functions of *B. terrestris *and related hymenoptera, mean that this EST library is a useful resource for ongoing research.

## Methods

### Animals

Colonies were set-up with field caught queens collected from Northern Switzerland in the spring of 2007 (Aesch, Basel), and were kept under red-light illumination at 27 ± 1°C with sugar water (ApiInvert^®^) and pollen provided *ad libitum*. Worker bees used for the EST library originated from four colonies (two workers each) that had been confirmed as *Bombus terrestris *by visual checks and based on a species-specific length polymorphism marker in the region between mitochondrial CO1 and CO2 genes. The colonies were also confirmed to be free of common parasites by microscopic investigation of faeces. Worker bees were age controlled, and one bee from each colony was 7 and one bee 14 days post adult eclosion at the time of sacrifice. Bees were sacrificed by snap freezing in liquid nitrogen, and were subsequently stored at -80°C. Tissue from both the thorax and abdomen was used as material for the EST library.

### RNA isolation and cDNA library construction

Total RNA was extracted with Solution D [58]. cDNA was synthesized using the SMART approach (Mint-Universal cDNA synthesis kit, Evrogen, Russia), subsequently normalized using duplex-specific nuclease (Trimmer kit, Evrogen, Russia) according to manufacturer's instructions, and directionally cloned into pAL32 plasmid vector (Evrogen, Russia). Plasmids were transferred via electroporation into *E. coli *DH10B (Invitrogen, U.S.A.).

### EST sequencing, quality control and assembly

Plasmids from the normalized libraries were 5' end sequenced using the pALforward primer (5'-CTCGGGAAGCGCGCCATT-3') and Big Dye Terminator chemistry (ABI). Collected reads from 3730XL capillary sequencers (ABI) were base-called using Phred http://www.phrap.org and subsequently quality and vector clipped using Lucy http://compbio.dfci.harvard.edu/tgi/software with standard parameters. For clustering and assembly, the TGI tools developed at TIGR http://compbio.dfci.harvard.edu/tgi/software were used.

### Annotation and comparisons

Where explicit packages and scripts are not mentioned, results were obtained by a mixture of custom Perl scripts implementing BioPerl modules [59] and custom R functions in R2.9.2 for Mac [60].

Once clusters were created, the resulting unique sequences were used for various comparisons. BLASTX searches with a cut-off of 1e-05 were run against each of the nr database in Genbank (version dated 19th October 2009), and databases comprising of all predicted proteins in the genomes of *A. mellifera*, *N. vitripennis*, *D. melanogaster *and *T. castaneum*.

Redundancy in the unique sequences was determined by comparison with *A. mellifera*. This was calculated as the number of unique *A. mellifera *proteins that were "best-hits" in the BLASTX search divided by the number of unique *B. terrestris *sequences with hits. GC-content was calculated using a custom Perl script that amalgamated all unique sequences and calculated the proportion of G and C bases in all unambiguous bases.

Annotation of GO terms [61] to the current *B. terrestris *EST derived unique sequences were carried out using the Perl based annot8r [38]. A database containing SwissProt and Trembl (Uniprot Knowledgebase Release 15.9) with manually curated GO terms was used. Terms that had been derived electronic annotation were omitted to improve reliability of the annotations. BLASTP against this database was carried out with a cut-off of 1e-05. In addition, predicted proteins from the entire genomes of *A. mellifera *[32] and *T. castaneum *[39] were compared with the same database for comparisons of the distribution of GO terms. For statistical comparisons, GO terms were matched to higher-level GO Slim categories. Pairwise comparisons between *B. terrestris *and both *A. mellifera *and *T. castaneum *were then carried out for the distribution of sequences in each GO Slim category using Fisher's exact tests in R2.92 for Mac [60].

The EST unique sequences were compared with the *A. mellifera *genome [32] using BLASTN, cut-off 1e-08. A custom Perl script was then used to extract positional information of all the "best-hits". Linkage group and position on the linkage group was then compared with the position of predicted protein coding regions within the genome to obtain a relative position of the "hits" (inside a protein-coding region; spanning a protein-coding region boundary; outside a protein coding region). For those outside a predicted protein-coding region, the distance to the closest was calculated. For those *B. terrestris *sequences with hits greater than 4'375 bases (the largest *A. mellifera *UTR, obtained from the UCSC genome browser [42]) away from a protein coding region, a custom Perl script was used to interface with ORF Finder http://www.ncbi.nlm.nih.gov/gorf to obtain ORF information. Of these sequences, the 20 with the largest ORFs were investigated manually. BLASTP searches were carried out against the nr database, and if the "best-hit" was to another insect protein (cut-off = 1e-20), the "best-hit" was taken and blasted (TBLASTN) against the *A. mellifera *genome. It was confirmed that the "hit" location was the same as for the EST derived unique sequence and the Blast alignments were manually examined for potential features inhibiting protein production.

### SSR and SNP marker identification

To identify potential genetic markers we used the Perl scripts MISA [50] and SNPidentifier [57] to locate simple sequence repeats and single nucleotide polymorphisms, respectively.

With MISA the configuration file was adjusted to identify motifs of two, three, four, five or six nucleotides, that repeat at least six times for di-nucleotide repeats and five times for all others. MISA was then run on all the post-assembly unique sequences. With SNP identifier we adjusted the minimum minor allele frequency to 0.1 and occurrence to 2. This meant that post-assembly sequences needed to be made up of at least four ESTs to be considered. SNPidentifier was run on the alignments of the EST sequences.

## Authors' contributions

BMS prepared the animals for the EST library, carried out bioinformatics analyses of the sequences. MK, SK and RR contributed to RNA isolation, library construction, template preparation, sequence determination and assembly. BMS, MK, SK, RR and PSH drafted the manuscript. PSH was the initiator of this project. All authors read and approved the final manuscript.

## Supplementary Material

Additional file 1 Assembly statistics of the *Bombus terrestris *ESTs. Click here for file

Additional file 2 Counts of the "best-hits" at the individual taxonomic identifier level for the 13,333 post-assembly *Bombus terrestris *EST sequences (BLASTX, cutoff = 1e-05). Click here for file

Additional file 3 Presence and identity of "best-hits" for the 13,333 unique *Bombus terrestris *sequences in the non-redundant (nr) database of Genbank (version dated 19th October 2009) (BLASTX cutoff = 1e-05). Click here for file

Additional file 4 Gene Ontology assignments for the unique *Bombus terrestris *sequences. Click here for file

Additional file 5 Presence and identity of "best-hits" in paired BLASTX comparisons (cutoff = 1e-05) of the unique *Bombus terrestris *sequences against proteins from each of *Apis mellifera*, *Nasonia vitripennis*, *Tribolium castaneum *and *Drosophila melanogaster*. Click here for file

Additional file 6 ***Bombus terrestris *****unique sequences with "hits" to proteins from genes characterised as immune related in *****Apis mellifera***** and *****Drosophila melanogaster***. Click here for file
