# Supplementary material for: Analysis of a normalised expressed sequence tag (EST) library from a key pollinator, the bumblebee Bombus terrestris
Source: BMC Genomics. 2010 Feb 15;11:110. doi: 10.1186/1471-2164-11-110 (PMC2838840; doi:10.1186/1471-2164-11-110)
Supplement: Additional file 1 — Assembly statistics of the Bombus terrestris ESTs. [file 1471-2164-11-110-S1.PDF]

***Bombus terrestris* EST and assembly statistics**

Total number of sequence reads	31,160
High quality sequences after filtering†	29,428
Average EST size after trimming (bp)	661
Total number of assembled sequences	13,333
Number of contigs	4,682
Number of singletons	8,651
Longest contig (bp)	3,542
Average contig length (bp)	960

† High quality sequences are those of at least 100bp after trimming vector sequences, removing cloning relics and contamination
